# Q-optimised nanoelectromechanical diamond resonators

**DOI:** 10.1038/s41378-026-01189-1

**Published:** 2026-03-03

**Authors:** Evan L. H. Thomas, Soumen Mandal, William G. S. Leigh, Oliver A. Williams

**Affiliations:** 1 School of Physics and Astronomy, Cardiff University, Queen’s Buildings, Cardiff, UK; 2 Now at Institute of Micro Production Technology, Leibniz University Hannover, Garbsen, Germany

**Keywords:** Structural properties, NEMS, Structural properties

## Abstract

Nanomechanical resonators are increasingly becoming of interest across a range of applied and fundamental physics applications. Within many of these, the retention of bulk diamond’s high Young’s modulus, coupled with the compatibility with standard substrate materials, makes nanocrystalline diamond (NCD) particularly well suited for fabricating high-frequency devices. As device dimensions shrink in pursuit of ever-higher frequencies, however, dissipation from sources such as clamping and surface loss often becomes increasingly significant. To address this, a series of doubly clamped beams and clamping-loss-suppressing free-free resonator geometries were fabricated from both as-grown and chemically mechanically polished NCD. At 12 K, the free-free geometries curtailed the pronounced length-dependent loss seen in doubly clamped beams, reducing dissipation by up to 8.8× and achieving *Q* factors of the order of 10,000 from ~40 MHz to ~100 MHz. Minor differences in dissipation between devices fabricated from the as-grown and polished stock, meanwhile, suggest that surface-related loss is likely a minor contributor to dissipation at this temperature, contrasting with trends in alternative material counterparts. As such, the combination of NCD’s apparent low surface-related loss and the loss-scaling suppression offered by free-free geometries provides a promising route to minimising dissipation in high-frequency nanomechanical resonators.

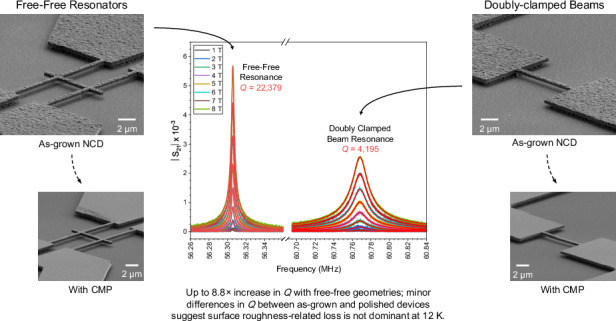

## Introduction

Micro- and nano-electromechanical resonators are increasingly used in a wide range of applications, from yoctogram-resolution mass sensors to demonstrating the applicability of quantum mechanics to macroscopic mechanical systems^1,2^. In many of these applications, high resonant frequencies and low dissipation (*Q*^−1^) are sought to enhance device performance. For instance, in mass sensing, achieving these conditions produces more detectable frequency shifts upon mass accretion^3^, while in quantum experiments, higher resonant frequencies raise the temperature at which ground-state cooling becomes possible^4^.

For doubly-clamped beams oscillating flexurally in the plane of the substrate, the resonant frequency is proportional to the product of the beam’s geometry, *w/L*^2^ (where *w* is the width and *L* is the length), and the material’s acoustic velocity, $\sqrt{E/{\rho }}$ (where *E* is the Young’s modulus and *ρ* is the mass density). Owing to its unrivalled acoustic velocity, diamond is thus well suited for the fabrication of high-resonant-frequency structures, with its use expected to yield resonant frequencies up to 1.6× and 2.4× higher than those of similarly dimensioned devices fabricated from silicon carbide and silicon, respectively^5^. However, while single-crystal diamond (SCD) offers exceptional material properties, it often requires unconventional handling and non-trivial mounting strategies that complicate integration with standard microfabrication processes^6^. In contrast, nanocrystalline diamond (NCD) retains this high Young’s modulus in a thin-film form that can be readily deposited atop widely used sacrificial layers, thus permitting the creation of high-frequency nano-mechanical diamond resonators without the integration challenges often associated with use of the material.

While choosing a structural material with high acoustic velocity can raise resonant frequency, substantial increases often necessitate alterations to device geometry, with resonator length usually adjusted first due to the *L*^−2^ dependence. Such changes, however, often increase dissipation, negating the potential gains in performance^7^. Specifically, significant strain at either end of the beam will subject the support anchors to shear forces and moments, in turn exciting elastic waves away from the device and into the bulk upon resonance^8^. The amount of dissipation that results depends on the device dimensions, the extent of overhang of the support structure, and the mode of actuation; for example, resonators affixed to a supporting structure of similar thickness with significant undercuts exhibit loss proportional to *(w/L)*^3^ when actuated in the plane of the film from which they are fabricated^8,9^. As such, shortening devices to the point where the aspect ratio (*L/w* for in-plane actuation) falls below 100 results in a dominant clamping loss^9,10^. To counteract such scaling in *Q* as higher operating frequencies are pursued, geometries that alter the acoustic impedance of devices can be used to prevent energy transmission into the anchors. Accordingly, positioning an array of shorter cantilevers along a central doubly clamped beam, inducing out-of-phase resonance of neighbouring resonators, and affixing torsional or flexural supports at nodal points of a beam to create ‘free-free’ clamping conditions have all been shown to reduce anchor loss. Examples of such are predominantly in resonators fabricated from silicon, with more limited demonstrations in silicon carbide and diamond^11–14^.

As dimensions continue to shrink in the pursuit of ever-increasing resonant frequencies, mechanical resonators are expected to become harder to actuate and detect, and increasingly susceptible to energy losses dominated by surface effects^15^. Sub-micron thick silicon cantilevers with sufficiently large aspect ratios exhibit dissipation that scales linearly with device thickness at room temperature, and thus surface-to-volume ratio, suggesting that loss is governed by surface properties rather than bulk characteristics^16,17^. Consequently, the removal of native and thin, fabrication-induced oxide layers has been shown to increase *Q* by up to an order of magnitude in silicon resonators, with this improvement subsequently reversed upon oxide regrowth at a rate that is proportional to layer thickness^17,18^. Rather contrarily, resonators coated with thermally or plasma-grown oxides have also demonstrated *Q* factors exceeding those of devices with comparatively thinner native or ozone-grown layers, suggesting that mechanical loss depends more on oxide quality than mere presence^18,19^. Further investigations into alternative passivation routes of the reactive silicon surface with hydrogen, methyl-, and longer-chain alkyl groups have demonstrated increases in *Q* exceeding those of the as-fabricated surface by up to an order of magnitude, often becoming more pronounced at cryogenic temperatures, as well as a dissipation that correlates more strongly with the density of electronically active surface defect sites than with the mechanical properties of terminating layer^20–22^. It was thus concluded that energy loss within such resonators primarily arises from coupling between the resonator’s strain field and localised states at the bulk-surface interface, with up to 75% of the dissipation observed within oxide-terminated resonators linked to the surface^20–22^.

Diamond’s lack of native solid-state oxide and largely stable surface chemistry meanwhile renders the material resistant to many such temporal degradations in *Q*. Nonetheless, dissipation in diamond devices does remain strongly influenced by surface functionalisation; annealing kilohertz-range SCD resonators to either oxidise Ar/Cl-based RIE-etched surfaces rich in H, O, and Cl species or to remove adsorbates from oxygen-terminated surfaces, has been shown to reduce loss by up to an order of magnitude at room temperature^23,24^. Upon cooling to cryogenic temperatures, bulk-related loss mechanisms then increasingly contribute, diminishing but not eliminating the influence of surface-related dissipation^23^. It then follows that materials in possession of significant surface roughness and increased surface-to-volume ratio could exacerbate surface-attributable loss, with such trends in dissipation observed in both macroscopic silicon cantilevers and nanoscale silicon carbide beams at cryogenic temperatures^14,25^. The significant surface roughness inherent to high Young’s modulus NCD films could therefore be expected to hamper efforts to achieve high-fQ product thin-film diamond NEMS^26^.

This paper thus attempts to investigate the effects of clamping and surface losses for NCD resonators through comparing the performance of doubly clamped and ‘free-free’ geometries fabricated from as-grown and chemically mechanically polished (CMP) nanocrystalline stock. Upon actuation of the resulting structures at cryogenic temperatures, clear differences in *Q* factor are observable between the two geometries with no observable dependence on the roughness, highlighting the dominant loss mechanisms under the testing conditions and the need for mitigation.

## Results

**Fig. 1:**
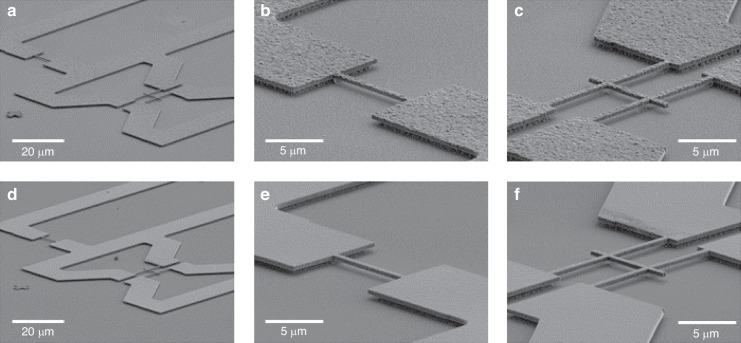
Tilted SEM micrographs of free-standing structures designed to resonate at ~100 MHz. Panels (**a**) and (**d**) show pairs of resonant structures fabricated from the as-grown and CMP polished stocks, respectively. Panels (**b**) and (**e**) then present magnified views of the corresponding doubly clamped beams, while panels (**c**) and (**f**) show the 'free-free' resonator geometries. The latter geometry incorporates second-order flexural supports to reduce dissipation attributable to clamping loss

Using the process detailed within the experimental methods, a series of doubly clamped beams and free-free resonator geometries were fabricated from as-grown and CMP-polished NCD stock, with chromium and gold capping layers for the purpose of actuation. Possessing nominal widths of 500 nm and nominal lengths between 8.8 μm and 13.95 μm, the devices are expected to generate resonant frequencies within the ~40 MHz to ~100 MHz range. Within the free-free design, the fixed clamps that anchor the main beam in the doubly-clamped geometry are replaced by pairs of flexural supports that are designed to resonate at a frequency corresponding to that of the second-order mode of the main beam^13^. These supports are then affixed at points corresponding to the zero-displacement nodes of both the supports themselves and the main beam, creating regions of high mechanical impedance that restrict energy transfer away from the main beam into the anchor supports, thereby limiting dissipation. Figure 1 contains tilted scanning electron microscope (SEM) micrographs of one such set of free-standing structures designed to resonate at ~100 MHz and fabricated from both the as-grown rough and CMP polished smooth stock (Panels a and d), along with magnified images of the respective doubly clamped (Panels b and e) and free-free resonator geometries (Panels c and f).

Upon in-plane magnetomotive actuation of the fabricated devices at pressures of ~10^−4^ mbar and a temperature of 12 K, 15 distinct resonances were observed and attributed to mechanical motion: 9 from the rough stock (5 doubly-clamped beams and 4 free-free resonators) and 6 from the smooth stock (3 doubly-clamped beams and 3 free-free resonators). For each resonance, three transmission coefficient measurements (S_21_) were taken at magnetic field strengths ranging from 0 T to 8 T in 0.5 T steps to incrementally increase the Lorentz force acting on the resonators. When plotting the imaginary part of S_21_ versus the real part, the response of magnetomotively actuated resonators is expected to trace a circle that intersects the real axis at both the origin and the resonant frequency^27^. However, finite background transmission introduces a complex translation that moves the circle away from this canonical position, while measurement-induced phase shifts rotate it about the origin^28^. These effects were first estimated by fitting the circle to provide initial parameter values, before performing a full fit utilising the expected Lorentz-like response of the resonators with the incorporation of additive terms to account for constant and frequency-dependent background contributions, and a multiplicative phase factor^29^.

**Fig. 2:**
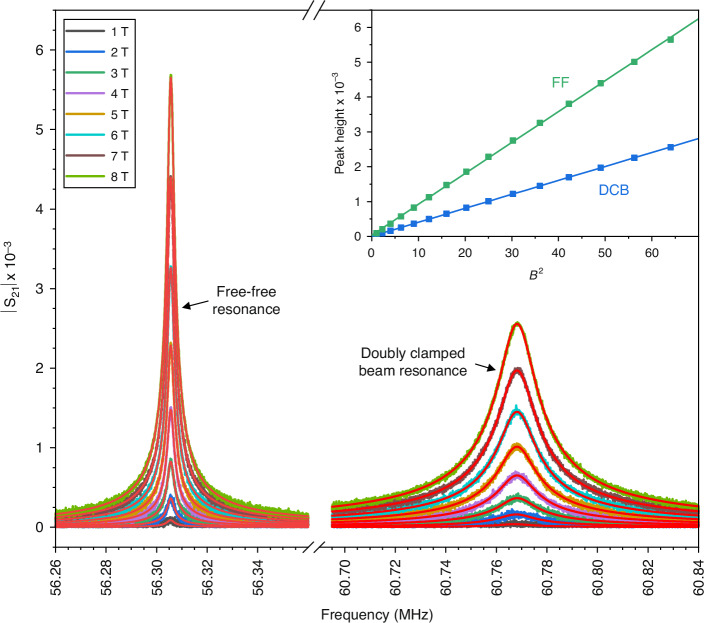
Transmission measurements of a ~60 MHz resonator pair. Main: Magnitude of the transmission coefficient measurements (|S_21_ |) obtained from one of the ~60 MHz resonator pairs fabricated from the rough stock after background removal, with the free-free (FF) resonator on the left and the doubly clamped beam (DCB) on the right, and the overlaid lines showing the result of the fitting process. Inset: resonance peak amplitudes versus the magnetic field strength squared, with the associated linear fit. The symmetric lineshape of the peaks and the linear relationship between peak height and B^2^ confirm that the signals observed originate from the mechanical resonance of the fabricated devices

Figure 2 plots a set of magnitudes of the de-embedded transmission coefficients for one of the pairs of ~60 MHz resonators fabricated from the rough stock with the background components removed. The resonant peak attributable to the free-free geometry appears on the left, and the peak attributable to the doubly clamped beam on the right. The overlaid lines detail the result of the fitting process, while the inset figure shows the fitted peak heights of the respective traces against the square of the magnetic field strength. For reference, Fig. S1 in the Supplementary Information plots the same ~60 MHz rough stock resonator traces prior to de-embedding. In the main plot of Fig. 2, the observed traces appear symmetric and are well matched by the fitted Lorentz-like profiles, suggesting that the device responses are linear for the applied drive powers^30,31^. In this linear regime, the force acting on the resonators, and hence both the displacement and velocity, is proportional to the applied magnetic field strength. The resulting motion of the conductive beam within the magnetic field then induces an electromotive force (EMF) that is proportional to the product of the field strength and the velocity, leading to a voltage that scales with B^2^ ^27,32^. The trends observed in the inset figure thus align with this behaviour and confirm that the measured voltages originate from the mechanical motion of the resonators rather than spurious electrical resonances within the measurement setup^14,33^.

**Fig. 3:**
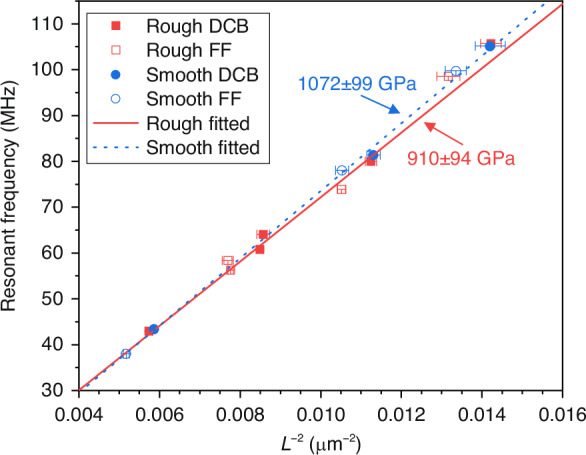
Frequency vs. length^−2^ scaling for the devices fabricated from the smooth and rough stocks with the associated linear fits in accordance with the Euler-Bernoulli beam equation. Young’s modulus values of 910 ± 94 GPa and 1072 ± 99 GPa are extracted from the linear fits for the rough and smooth stock devices, respectively, while incorporating uncertainties in the fit, device length, device width, and diamond layer thickness

The dependence of the resonant frequency on the SEM-measured device length for the full sample set is then plotted in Fig. 3. As visible in the plot, the extracted resonances exhibit a clear *L*^−2^ dependence, in accordance with the Euler–Bernoulli theory-derived equation that governs the relationship between resonant frequency and device dimensions^34^. The overlaid lines represent the result of fitting after grouping the samples by the wafer from which they were fabricated. In the case of out-of-plane flexural and torsional cantilevers, the inevitable undercut when utilising an isotropic-etch release step reduces clamp stiffness and increases the effective length. This effect, which depends on the length of the undercut and the resonance mode shape, is expected to cause a downshift in the resonant frequency^34–36^. While the undercuts visible within Fig. 1 could therefore be expected to perturb the resonant frequencies of the in-plane free-free geometries and doubly clamped beams to different degrees due to their distinct boundary conditions, the devices were nevertheless grouped to increase the number of data points and the robustness of the linear fits^37^.

With the added mass and stiffness of the metallic electrodes on top of the devices, the resonant frequency (*f*_*0*_) of the composite beams can be expressed as:

$${f}_{0}=\eta \sqrt{\frac{{E}_{d}{I}_{d}+{E}_{c}{I}_{c}+{E}_{g}{I}_{g}}{{\rho }_{d}{A}_{d}+{\rho }_{c}{A}_{c}+{\rho }_{g}{A}_{g}}}{L}^{-2}$$

where *E*_*i*_, *I*_*i*_, *ρ*_*i*_, and *A*_*i*_ are the Young’s modulus, moment of inertia, density, and cross sectional area, respectively, of the diamond (d), chromium (c), and gold (g) layers^38,39^. The term *η* is a numerical factor equal to 3.57 for the fundamental mode of a doubly clamped or free-free beam. Prior to release, the mean thicknesses of the rough and smooth device sample sets, including the chromium and gold layers, were determined with atomic force microscopy (AFM) to be 442.0 ± 0.4 nm and 444.9 ± 0.1 nm, respectively. Incorporated in these values are the nominal deposition thicknesses of 10 nm for chromium and 45 nm for gold. Based upon the gradients within Fig. 3, and utilising the mass density of bulk diamond of 3.5 g cm^−3^ along with the aforementioned thickness values, Young’s modulus values of 910 ± 94 GPa and 1072 ± 99 GPa are then extracted for the rough and smooth stock diamond films, respectively^40^. The discrepancy between these values is likely due to the small number of samples used in the fitting process and minor device-to-device variation. Both extracted Young’s modulus values, meanwhile, align well with those obtained from actuated micro-resonators, in which similarly large undercuts at the clamping structures effectively lengthen the beam, leading to underestimates of the constituent material’s Young’s modulus^30,33,36,41,42^. While the presence of sp^2^-hybridised carbon and hydrogen within the grain boundaries of polycrystalline diamond can significantly reduce the Young’s modulus from that of bulk diamond, with more modest effects on mass density, similarly synthesised films have previously demonstrated values of 1100 GPa when characterised via the pressure-induced deflection of free-standing membranes^26,43–46^.

**Fig. 4:**
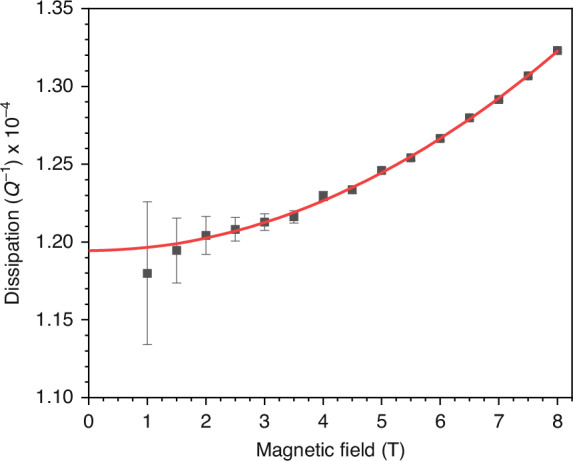
Dissipation (Q^−1^) vs. magnetic field strength for the ~40 MHz doubly clamped beam fabricated from the smooth stock with associated quadratic fit. The observed increase in dissipation with magnetic field strength is well characterised by magneto-motive damping, with the y-axis intercept indicating an unloaded dissipation of 1.19 × 10^−4^, corresponding to a quality factor (Q) of 8370

As the fabricated structures oscillate, energy will be lost to the surrounding environment. This dissipation is quantified by the inverse of the quality factor, *Q*^−1^, where *Q* = *f*_*0*_/∆*f* and ∆*f* is the full width at half maximum power, or equivalently, $1/\sqrt{2}$ of the maximum signal amplitude of the Lorentzian-like resonance profiles shown in Fig. 2. When using the magnetomotive transduction scheme, the finite impedance of the measurement circuitry allows the EMF generated by the motion of the resonator to drive an additional current, which interacts with the magnetic field to produce a Lorentz force that opposes the beam’s velocity. The magnetomotive loss that results then scales with *Q*^−1^ *∝* *B*^2^*L*^4^, leading to a quadratic increase in dissipation with field strength, with the magnitude decreasing as device length shortens^7^. The unloaded dissipation of such actuated resonators can therefore be determined by measuring the loaded dissipation as a function of magnetic field and extrapolating to zero field^33,47^. Figure 4 depicts the dissipation as a function of magnetic field strength for the 13.95 μm long, ~40 MHz doubly clamped beam fabricated from the smooth stock, along with the associated quadratic fit. While the fitting was performed using the three values of dissipation obtained at each field strength as individual data points, these values have been averaged and their associated errors combined within the figure for clarity. The unloaded dissipation at zero field strength for the device is then extracted to be 1.19 × 10^−4^, corresponding to a Q factor of 8370.

**Fig. 5:**
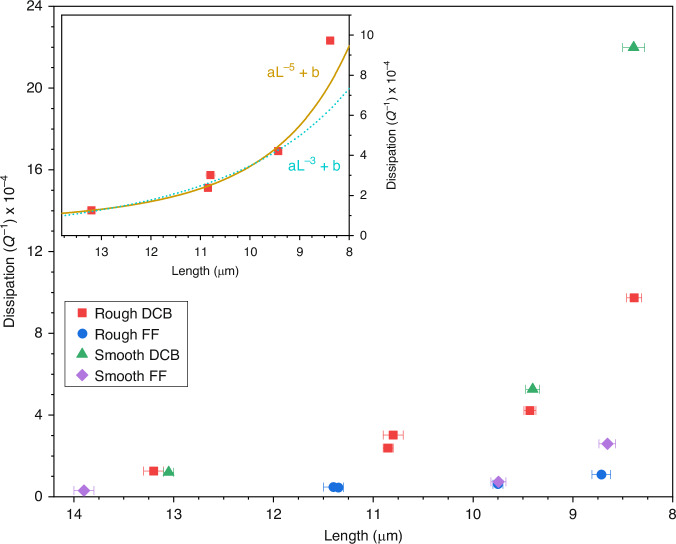
Dissipation (Q^−1^) vs. length for the devices fabricated. Main: Dissipation Q^−1^ vs. length for the full set of doubly clamped beams and free-free resonator geometries fabricated from the rough and smooth stock. Inset: Dissipation Q^−1^ vs. length for the doubly clamped beams fabricated from the rough stock with associated fit to aL^−3^ + b and aL^−5^ + b. Reductions in the length-dependent dissipation are observed upon the addition of the second-order flexural supports of the free-free geometry, indicative of a reduction in clamping losses

Figure 5 plots the unloaded dissipation as a function of device length for the entire sample set, while the inset figure plots the dissipation solely for the doubly-clamped beams fabricated from the rough stock. The error bars visible in both plots represent the error in device length obtained from repeated measurements of device length with SEM. Also shown in the inset figure are the results of fitting the dissipation data to the functions *aL*^−3^ + *b* and *aL*^−5^ + *b*, where *a* and *b* represent the coefficients of the length-dependent and length-independent loss mechanisms, respectively. Although the dataset is limited and spans a narrow length range, the *L*^−5^ scaling tentatively appears to provide the best fit, consistent with previously observed behaviour in similarly constructed 10–19 μm long NCD resonators, with the length-dependent contribution to dissipation attributed to clamping loss^47^. This scaling suggests more rigid clamping than the *Q*^−1^ *∝ (w/L)*^3^ scaling predicted for in-plane resonators with supporting structures of similar thickness to the resonator itself^8^. Similar behaviour has been reported for out-of-plane actuation, where clamping loss scales as *L*^−1^ when supported by thin, pliant plates, and as *L*^−5^ when mounted on thick, rigid substrates, illustrating that increased clamping stiffness weakens the dependence of dissipation on device length^48,49^. Upon incorporating second-order flexural supports to form the free-free geometry, the increase in dissipation with decreasing length is significantly reduced, with the *a*-coefficient for the *L*^−5^ fit dropping by close to an order of magnitude from 29.3 ± 3.5 (μm)^5^ to 3.8 ± 0.8 (μm)^5^. The length-independent offset *b* terms are meanwhile 5.2 ± 2.2 × 10^−5^ and 2.2 ± 0.9 × 10^−5^ for the doubly clamped beam and free-free geometries, respectively. This difference in length-dependent dissipation leads to dissipation values of 9.7 × 10^−4^ and 1.1 × 10^−4^, or an 8.8× reduction, for the ~100 MHz, 8.8-μm nominal length resonator pair fabricated from the rough stock. Devices fabricated from the smooth stock meanwhile show similar dissipation trends to their rough stock counterparts, with an initial dissipation that is marginally lower for the ~13 μm, ~40 MHz devices, before the dissipation increases beyond that of the rough stock devices, with the disparity growing as device length reduces. This then results in dissipation values of 2.2 × 10^−3^ and 2.6 × 10^−4^ for the ~100 MHz doubly clamped beam and free-free geometry pair, corresponding to an 8.5× reduction. Fitting to the trends observed from the smooth stock devices was also carried out; however, the further reduced number of devices limited the robustness of the fit, rendering the results indicative rather than definitive. The obtained *a* coefficients are 41.6 ± 16.1 (μm)^5^ and 4.9 ± 1.9 (μm)^5^, and the *b* coefficients 0.9 ± 5.3 × 10^−5^ and 2.1 ± 1.3 × 10^−5^ for the doubly clamped beam and free-free designs, respectively. While possessing large uncertainty, the *a* values correspond to similar reductions in clamping-loss as the rough counterpart values, albeit more pronounced, and the *b* values indicate no statistically significant difference in the length-independent loss observed.

## Discussion

When attributing dissipation to specific loss mechanisms in nano-electromechanical resonators, many can be excluded for the current sample set based on their geometries and the specifics of the characterisation setup. At temperatures of 12 K, thermoelastic dissipation arising from localised temperature variations at a frequency mismatched to the thermal relaxation time of the constituent NCD is expected to contribute less than 10^−12^ to the total dissipation^50^. The ~10^−4^ mbar pressures under which the devices were tested are, meanwhile, orders of magnitude below the pressure threshold at which molecular damping becomes relevant for structures of the proportions fabricated^7^. Additionally, internal losses in the gold and chromium comprising the metal electrodes are estimated to account for <7% of the observed dissipation, with the largest contribution seen for the device that exhibits the lowest dissipation, the ~40 MHz free-free beam fabricated from the smooth stock^51,52^.

With the aspect ratio of the devices fabricated, dissipation is rather expected to be dominated by clamping loss. As dictated by the boundary conditions, strain concentrates at the interface between the resonator and its supporting structure, allowing elastic energy to leak into the clamps and resulting in a loss that scales with *α(w/L)*^3^, where *α* depends on the Poisson’s ratios of the constituent materials and the mode order of the resonator^8,9^. The strong length-dependent dissipation observed for the doubly clamped beam fabricated from the rough stock, shown in the inset of Fig. 5, is consistent with such loss. The addition of second-order composite supports at the respective zero displacement nodes of both main beam and supports to form the free-free geometry then significantly suppresses this length-dependent dissipation^13^, with a measured 8.8× reduction at ~100 MHz. This is comparable to reductions reported for polysilicon and silicon carbide resonators over the 10 MHz to 180 MHz range^12–14^. The residual length-dependent dissipation in the free-free resonators fabricated from the rough stock is then likely due to the finite widths of the supporting tethers and resulting imperfect nodal anchoring, fabrication-induced dimensional variations, and discrepancies between the estimated and actual material properties. Prior simulations of free-free geometries have suggested minor changes in *Q* are expected upon small deviations in the placement of the supporting structure from the nodal locations^53,54^. With clamping losses expected to affect both the rough and smooth stock devices similarly, the resonators fabricated from the smooth stock display a similar length dependency, albeit increasingly pronounced at shorter lengths. This difference in length-dependent dissipation is likely due to challenges in achieving consistent undercut depths during device fabrication, which directly impact clamping loss and device-to-device variation of the nano-scale features. Both the formation of an etch-resistant silicon carbide layer during diamond deposition and temperature non-uniformities during etching are expected to influence the rate and uniformity of HF vapour etching of the thermally grown SiO_2_ sacrificial layer^55,56^.

Since surface-related dissipation is expected to be largely independent with respect to resonator length, the lack of resolvable difference in the length-independent contribution to the dissipation observed in devices fabricated from the rough and smooth stock suggests that surface loss associated with the upper surface of the resonators is not the dominant form of dissipation at 12 K. This contrasts with behaviour observed in silicon carbide beam resonators at 4.2 K, where devices fabricated from stock with roughness ≤2 nm operated into the UHF/microwave range, while those from rougher stock (up to 7.1 nm roughness) showed significantly degraded *Q*-factors, limiting their operation to the VHF range^14^. Silicon cantilevers possessing lengths in the mm-range and thicknesses of the order of 100 μm have meanwhile exhibited a mechanical loss that is largely independent of surface quality at room temperature, but strongly correlated with roughness of both the upper and lower surfaces at temperatures below ~100 K^25^.

In the case of diamond, prior studies have shown that high-aspect-ratio kHz-range cantilevers fabricated from optical-grade SCD exhibit loss that scales linearly with decreasing thickness, and hence increasing surface-to-volume ratio, indicative of sizeable surface-related dissipation at 300 K^23^. This behaviour aligns with observations in single-crystal silicon analogues^16,17^. Accordingly, oxidation of the disordered as-released diamond surface, rich in covalently bound H, O, and Cl species, to produce a cleaner and more defined termination has been shown to yield up to a ~25× reduction in dissipation^23^. At cryogenic temperatures, however, surface-related loss becomes less pronounced, with the reduction in dissipation achieved through oxidation diminishing to ~3.5×^23^. Similar temperature-dependent trends in surface-attributable loss have been observed in MHz-range SCD annular plate resonators, in which dissipation decreases upon cooling from 300 K and saturates near 60 K^57^.

As cooling continues and the differences in dissipation with surface treatment diminish, loss within disordered and highly miniaturised resonators often becomes dominated by defects that switch between equilibrium and metastable states, either via thermal activation or quantum tunnelling^7^. The combined response of many such defects, each with distinct activation energies and relaxation times, results in a loss that typically scales as *T*^*α*^. Prior studies on columnar NCD resonators have revealed a resolvable Debye peak at 35 K to 55 K, superimposed on a broader *T*^0.2^ trend extending from 2 K to 100 K^51,58^. As expected, increases in structural disorder and defect density then yield corresponding increases in dissipation, with cantilevers fabricated from electronic-grade SCD, optical-grade SCD, and 3–5 nm grain-size ultra-NCD exhibiting losses of roughly 10^−6^, 10^−5^, and 10^−4^ at 20 K^23^. Importantly, this latter value is an order of magnitude greater than the differences in loss observed at cryogenic temperatures after oxygen termination of the as-released surfaces in similarly thick optical-grade SCD cantilevers^23^. Thus, dissipation in the free-free geometries fabricated in the current work is likely to primarily originate from structural and chemical imperfections located either within the bulk or on the O_2_-etched and nucleation-underside surfaces common to both rough and smooth devices, residual clamping loss, or combinations thereof. While the underside of the resonators likely possesses roughness values lower than those of the polished top surface (prior studies of NCD films grown with seeding densities ≥10^10 ^cm^−2^ have exhibited values below 1 nm RMS), the large surface-to-volume ratio of the early-growth phase has previously been suspected to hamper *Q*, supporting the suggestion of bulk-derived loss^59–61^. Further characterisation of the temperature dependence of dissipation in a measurement setup that decouples pressure from temperature would likely enable more accurate determination of the dominant loss mechanisms in the smooth and rough NCD resonators fabricated.

When comparing resonator performance, the product of the resonant frequency and the *Q*-factor, *fQ*, is often used as a figure of merit. In this work, the highest *fQ* product measured for the doubly clamped beams was 3.63 × 10^11 ^Hz, observed for the ~40 MHz device fabricated from the smooth stock. This value compares favourably to similarly characterised MHz-range GaAs and Si resonators at 12 K, with reported *fQ* products of ~7 × 10^10 ^Hz and 1.2 × 10^11 ^Hz, respectively. The differences in *fQ* products between these values and those of the NCD resonators fabricated within correlate with the acoustic velocities of each material, highlighting the increase in resonant frequency achievable when transitioning to diamond without the aggressive down-scaling that typically introduces additional loss^62^. This value is also consistent with those reported for similar NCD-derived doubly clamped beams, which are often characterised at sub-kelvin temperatures far below the 12 K used within the current study, and is comparable to bulk-mode NCD disk resonators, which offer lower surface-to-volume ratios and often reduced surface loss, although the disks were characterised at 300 K^5,33,38,51,63^. Despite the higher defect density expected in NCD, this value also exceeds those reported for SCD cantilevers at cryogenic temperatures by an order of magnitude or more, highlighting the advantages of using thin-film diamond on an appropriate sacrificial layer^23,64^. The reduced dissipation enabled by the flexural supports in the free-free geometry further enhances the *fQ* product to values of the order of 10^12 ^Hz for all but the ~100 MHz devices, with the highest value of 1.26 × 10^12 ^Hz observed for the ~60 MHz device fabricated from the rough stock. Given the differing characterisation temperatures utilised (0.04 K to 1.1 K), direct comparison of these results to the low-10^13^ Hz *fQ* products obtained from the 400 MHz to 1440 MHz clamping loss mitigating NCD coupled-beam geometries fabricated elsewhere is challenging^11^. However, doubly clamped NCD beams have previously shown improvements in *Q* by a factor of ~4 upon reductions in temperature from 10 K to the mK range, suggesting that the *fQ* products of the present devices may also benefit from further cooling.

## Conclusion

A series of in-plane doubly clamped beams and free-free resonators were fabricated from as-grown and CMP NCD. Magnetomotive measurements at 12 K revealed distinct Lorentzian-like resonance peaks in the ~40 MHz to ~100 MHz range, with *B*^2^ scaling of both peak height and dissipation confirming their mechanical origin. Across the doubly clamped sample sets, clear length-dependent trends in dissipation were observed, consistent with clamping loss being the dominant form of dissipation. Introducing second-order flexural supports affixed at nodal points to create free-free geometries then substantially suppressed this clamping-related dissipation, resulting in an up to 8.8× reduction in dissipation at ~100 MHz and a *Q* factor of 9176, yielding *fQ* products as high as 1.26 × 10^12^ Hz. No clear difference in the length-independent dissipation between the as-grown and polished sample sets was observed, suggesting that surface-related loss is not the primary contributor to the dissipation of such NCD resonators at cryogenic temperatures, with loss likely being dominated by contributions from the bulk. Together, these findings highlight the potential of combining NCD and free-free geometries within high-frequency, low-loss nanomechanical resonators.

## Methods

Silicon (100) FZ wafers of 2-inch diameter and 500 μm thickness with 500 nm thermal oxide on both sides were used as substrates throughout. Prior to growth, the substrates were subjected to an oxygen plasma (30 W, 30 SCCM O_2_) for 1 min in a Plasma Etch PE-25 plasma cleaner to remove any organic contamination and promote the formation of a hydroxyl-rich surface. The substrates were then seeded through immersion in a dispersed nano-diamond/DI water colloid and placed in an ultrasonic bath for 10 min, with electrostatic attraction between the oppositely charged hydrogenated diamond particles and the SiO_2_ surface shown to yield particle densities of the order of 10^11 ^cm^−2^ ^65^. After spinning dry, the wafers were subsequently placed in a Seki AX6500 MWCVD reactor, where NCD film growth was carried out at power and pressure setpoints of 4.1 kW and 40 Torr, respectively, resulting in growth temperatures of ~790 *°*C as judged by in-situ dual wavelength pyrometry. An initial methane fraction of 5% CH_4_/H_2_ was used during the ‘incubation’ phase of growth to stabilise the seeds until a film thickness of ~50 nm was reached, as indicated by in-situ laser interferometry, before reducing to 1% CH_4_/H_2_ for the remainder of growth in order to realise high Young’s modulus NCD^26,66^. A ‘thick’ and ‘thin’ wafer were produced through monitoring the growth with the aforementioned laser interferometry and halting the process upon reaching the target thickness, with thicknesses at the centre of each wafer measured post-growth through the use of a Filmetrics F-20 spectral reflectance system at 549 nm and 409 nm, respectively.

To study the effect of surface roughness on *Q* factor, the thick wafer was then chemically mechanically polished with an oxalic acid modified colloidal silica polishing slurry to an indicated thickness of 413 nm^67^. Roughness values at the centre of the ‘rough’ as-grown and ‘smooth’ polished wafers were determined through AFM to be 34 nm and 3 nm RMS over a 25 μm^2^ area, respectively. Both wafers were then submerged in HF acid to remove any residual tightly bound silica on the polished sample and ensure consistent processing between each sample, before being diced into pieces suitable for further processing. Central pieces from each wafer were then taken and treated with piranha solution (1:3 H_2_O_2_:H_2_SO_4_ for 5 min) in an attempt to ensure a consistent oxygen termination between the samples and enhance adhesion of metal layers deposited in subsequent processing steps.

Electron-beam lithography was next carried out with a Raith eLine tool utilising a 410 nm thick MMA/PMMA bilayer, followed by thermal evaporation of a 105 nm thick tri-layer consisting of chromium, gold, and nickel using an Edwards E306A coating system. Within this stack, chromium acts as an adhesion layer, gold provides the conductive path for actuation, and nickel serves as an etch mask. Following lift-off, the patterned etch mask was used to selectively and anisotropically etch the NCD film with an Oxford Plasmalab 100 ICP operating at 10 mTorr chamber pressure, 40 SCCM O_2_ gas flow, 1500 W ICP power, 100 W RIE power, and a carrier wafer temperature of 25 °C. The metal etch mask was then removed in FeCl_3_, and the devices released from the SiO_2_ buffer layer using an Idonus HF vapour phase etcher with the carrier wafer held at a temperature of 50 °C.

Using this process, pairs of doubly clamped beams and ‘free-free’ resonator geometries were fabricated with nominal widths of 500 nm and lengths of 13.95 μm, 11.4 μm, 9.85 μm, and 8.8 μm. Slight, deliberate variations in the lengths within each pair were incorporated to ensure two distinct and easily attributable resonant responses. Based on the weighted average of the densities and Young’s moduli of diamond, chromium, and gold, the structures fabricated are expected to exhibit resonant frequencies centred around 40 MHz, 60 MHz, 80 MHz, and 100 MHz, with separations within each pair of 4 MHz to 5 MHz. The supporting structures of the ‘free-free’ geometries were designed in accordance with Hsu et al., with the nominally 500 nm wide flexural supports at either side of the main beam designed to resonate together at a frequency corresponding to that of the second-order mode of the main beam, and affixed at zero-displacement nodes to limit energy transfer^13^.

**Fig. 6:**
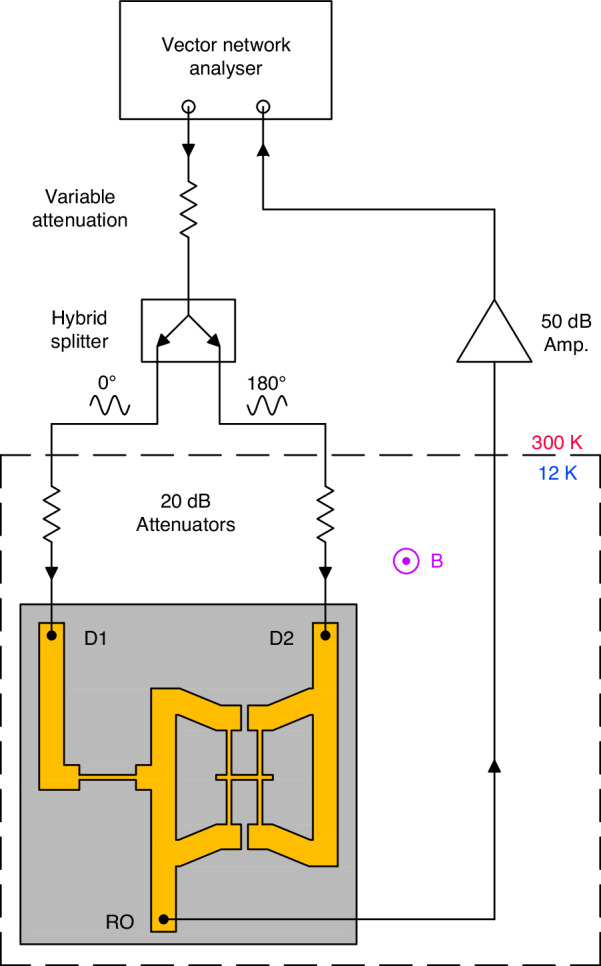
Schematic of the measurement setup used to characterise the fabricated devices. The devices were actuated magnetomotively within a Quantum Design PPMS at pressures of ~10^−4^ mbar and magnetic fields of up to 8 T, with the balanced bridge transduction scheme employed to suppress the large background arising from the static impedances of the metallised devices

After fabrication, the resistance of the devices at room temperature from bond pad to bond pad was measured through the use of a probe station equipped with a source measure unit to be of the order of 200 Ω. Characterisation of the resonant properties of the devices was then carried out in a Quantum Design Evercool physical property management system (PPMS) using the magneto-motive transduction scheme at magnetic fields up to 8 T^32^. A full schematic of the measurement setup is shown in Fig. 6. In this approach, the samples were mounted perpendicular to the magnetic field on a purpose-built, dipper-style probe equipped with RF lines, radiation baffles, thermalised attenuators, and an activated charcoal sorption pump. Prior to cooldown, the vacuum chamber was purged with helium repeatedly to remove residual atmospheric gas. Upon cooling, the temperature uniformity across the probe was monitored using a calibrated Cernox thermometer affixed beneath the sample stage, and a calibrated diode attached to the attenuator stage. Characterisation temperatures were fixed at 12 K to allow the sorption pump to reach pressures of ~10^−4^ while ensuring uniform thermalisation of the custom-built probe. A Rohde & Schwarz ZND vector network analyser (VNA) was then used to drive the devices with a swept-frequency signal, with the applied power varied between each set of devices to enhance the resulting signal while maintaining a linear response^30^. As the drive signal propagates along the resonators, a Lorentz force is generated, displacing the beam in the plane of the film and creating an EMF field that attenuates transmission through the device. The resulting signal was subsequently amplified at room temperature by a Cosmic Microwave Tech CITLF1 low-noise amplifier situated outside of the PPMS before feeding back to the input port of the VNA. To enhance the resonance signal, the balanced bridge technique was used in which two 180˚ out-of-phase voltages are applied to two back-to-back resonators (D1 and D2), nulling the background from the large static impedance of the metallised devices at the common read-out (RO) port^68^.

## Supplementary information

Supplementary Information

## Data Availability

Information on the data underpinning this publication, including access details, can be found in the Cardiff University Research Data Repository at 10.17035/cardiff.29994682.
