# Supplementary material for: Q-optimised nanoelectromechanical diamond resonators
Source: Microsyst Nanoeng. 2026 Mar 3;12:74. doi: 10.1038/s41378-026-01189-1 (PMC12953594; doi:10.1038/s41378-026-01189-1)
Supplement: Supplementary file 1 — Supplementary Information [file 41378_2026_1189_MOESM1_ESM.docx]

Supplementary Information for “Q-optimised nanoelectromechanical diamond resonators” by E.L.H. Thomas, S. Mandal, W.G.S. Leigh, O.A. Williams

*
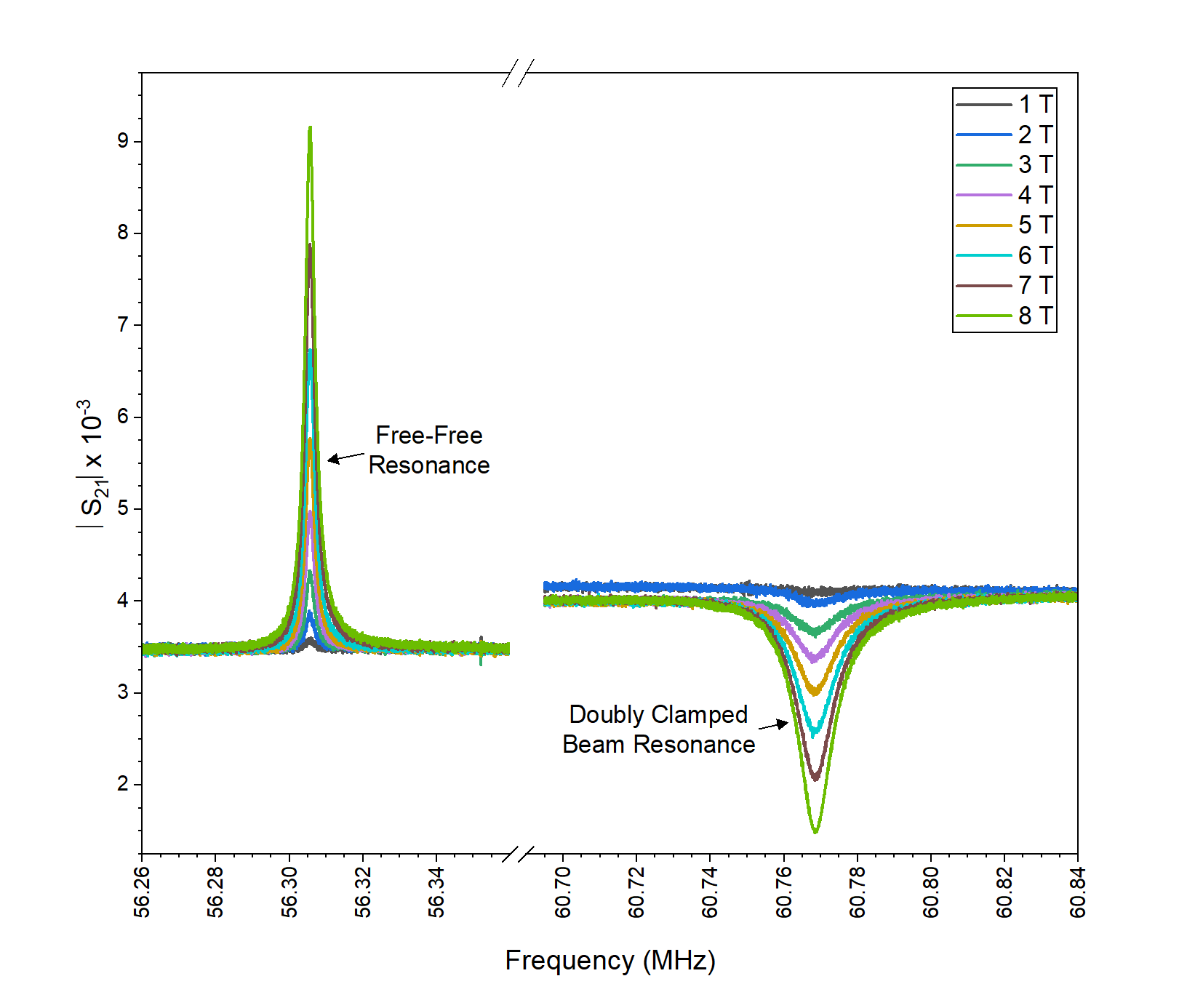
*

**Figure S1** Magnitude of the transmission coefficient measurements (|S_21_|) obtained from a ∼60 MHz resonator pair fabricated from the rough stock prior to background de-embedding, with the free-free resonator on the left and the doubly clamped beam on the right. After removal of the background transmission and measurement-induced phase shifts, the resonant peaks can be extracted to yield the de-embedded traces shown in Figure 2 of the main text.
